# Food Is Medicine for Individuals Affected by Homelessness: Findings from a Participatory Soup Kitchen Menu Redesign

**DOI:** 10.3390/nu15204417

**Published:** 2023-10-18

**Authors:** Marianna S. Wetherill, Lacey T. Caywood, Nicholas Hollman, Valarie P. Carter, Joshua Gentges, Ashli Sims, Carrie Vesely Henderson

**Affiliations:** 1 Department of Health Promotion Sciences, Hudson College of Public Health, University of Oklahoma Tulsa Schusterman Center, Tulsa, OK 74135, USA; 2 Department of Family and Community Medicine, University of Oklahoma School of Community Medicine, Tulsa, OK 74135, USA; 3 OU Culinary Medicine Program, University of Oklahoma School of Community Medicine, Tulsa, OK 74135, USA; valarie-carter@ouhsc.edu; 4 Office of Research Development and Scholarly Activity, University of Oklahoma School of Community Medicine, Tulsa, OK 74135, USA; nicholas-hollman@ouhsc.edu; 5 Department of Emergency Medicine, University of Oklahoma School of Community Medicine, Tulsa, OK 74135, USA; joshua-gentges@ouhsc.edu; 6 Iron Gate, Tulsa, OK 74103, USA; ashli@buildintulsa.com (A.S.); chenderson@irongatetulsa.org (C.V.H.)

**Keywords:** food insecurity, homelessness, food is medicine, culinary medicine, soup kitchens, nutrition, fruits and vegetables

## Abstract

Health disparities among people experiencing homelessness are likely exacerbated by limited access to healthy, fresh, and minimally processed foods. Soup kitchens and shelters serve as essential food safety nets for preventing hunger in this population, and community interest is growing in the potential of “food is medicine” interventions to improve the mental and physical wellbeing of people who receive meals from these providers. This study describes our two-phase approach to first identify and prioritize nutrition needs within an urban soup kitchen community and then test and implement new recipes and menu guidelines to help the standard soup kitchen menu better align with those priorities. We began by first conducting a nutrition needs assessment, including a collection of intercept surveys from a convenience sample of soup kitchen guests to better understand their nutrition-related health needs, dental issues, food preferences, and menu satisfaction (n = 112), as well as a nutrition analysis of the standard menu based on seven randomly selected meals. Most respondents reported at least one chronic health condition, with depressive disorders (50.9%) and cardiovascular diseases (49.1%) being the most common. Nearly all guests requested more fruits and vegetables at mealtimes, and results from the menu analysis revealed opportunities to lower meal contents of sodium, saturated fat, and added sugars and to raise micronutrient, fiber, and omega-3 content. We then applied these nutrition needs assessment findings to inform the second phase of the project. This phase included the identification of new food inventory items to help support cardiovascular and mental health-related nutrition needs, taste test sampling of new healthy menu items with soup kitchen guests, and hands-on culinary medicine training to kitchen staff on newly-developed “food is medicine” guidelines to support menu transformation. All taste tests of new menu items received over 75% approval, which exceeded satisfaction ratings of the standard menu collected during the phase 1 needs assessment. Findings from this community-based participatory research project confirm the great potential for hunger safety net providers to support critical nutrition needs within this vulnerable population through strategic menu changes. However, more research is needed on the longitudinal impacts of such changes on health indicators over time.

## 1. Introduction

The loss of housing severely compromises many dimensions of health, spanning nearly all aspects of mental, physical, and social wellbeing. Housing loss initiates a series of other basic need losses that further conspire against health, including limited choices of what, when, how, and where to eat. People without homes experience a six-fold increased risk for food insecurity, which may contribute to chronic disease and mental health inequities [1]. Limited food choices likely contribute to multiple nutritional risks identified in unsheltered populations, including inadequate micronutrient intake and low consumption of core food groups, such as fruits and vegetables [2]. Food insecurity may also play a role in hospitalization risk and emergency department use resulting from poorly managed chronic disease [1,3].

Soup kitchens and shelters play essential roles within the US food safety net system in their work to meet basic food needs of people without homes. However, due to the partial or full reliance on donated foods that can result in dramatically fluctuating food inventories, it is plausible that meals provided through many of these safety net providers lack essential nutrients and contain excessive nutrients linked to chronic disease exacerbation (e.g., sodium or added sugars). Little research is available to understand the scope and depth of this potential problem. An assessment of free meals served at different service providers around San Francisco, CA, found meals provided an average of 37% of calories from fat, exceeded recommended sodium content, and were inadequate in daily recommended amount of fiber (less than 30%), potassium, calcium, vitamin A, and vitamin E [4]. A similar study analyzing 41 meals from three soup kitchens in Grand Rapids, MI, found that the average meal fell below two-thirds of the recommendations for vitamin C, magnesium, zinc, and calcium, while exceeding recommendations for saturated fat [5]. Researchers found that while two daily meals generally met recommendations for all nutrients except fiber, these meals also exceeded calorie and saturated fat requirements and contained more than twice the amount of the daily recommended sodium [5]. Micronutrient adequacy for some nutrients (B vitamins, iron, and phosphorous) was attributed to the high use of enriched flour products [5], which is consistent with donated foods commonly found within the charitable food sector, such as white breads, pasta, and pastries [6]. One older study involving a nutrition analysis of foods consumed by women experiencing homelessness found average intakes below two-thirds of the Recommended Daily Allowance (RDA) for many nutrients, including vitamin B6, calcium, and iron [7].

Building on recent precedent efforts to provide medically tailored foods in food pantry settings [8], shelters and soup kitchens may also be novel sites for reducing housing-related health disparities through improved nutrition, including one recent demonstration project in Boston, MA [6]. Despite serving populations with complex health needs, the nutritional guidance for shelter and soup kitchen meal programs is lacking [6]. The complex physical and mental health issues within unsheltered populations warrant the development of specialized menus that are not only responsive to nutrition needs, but also likely to be consumed. Unsheltered individuals are more likely to report dental problems, which may affect the ability to eat a varied diet and achieve nutritional goals for health [9]. Modified textures may be an important consideration when planning a menu to address nutrient gaps. Further, shelters and soup kitchens may only supply 1–2 hot meals per day, indicating a need to maximize the nutrient density of these meals. For those organizations that supply take-away meals, additional planning considerations are necessary, including foods that are safe to eat later without the need for refrigeration.

Individuals experiencing homelessness may perceive or define their nutrition needs differently than other populations based on their past life experiences and living environment, yet frequently lack the opportunity to have a choice in the meals and types of food they consume. Informed by the definition for health equity [10], nutrition equity can be described as the absence of avoidable and unfair differences in nutritional intake and in the health outcomes perpetuated by these differences [11]. Thus, efforts to improve nutrition equity seek to improve individual and community access to food-related knowledge, expression, production, access, and utilization. Centering nutrition equity in food is medicine research requires the use of a participatory framework that involves intended users at all stages of intervention design and implementation.

This paper describes the participatory processes used to plan and implement a “food is medicine” menu redesign at an urban downtown soup kitchen. The goals of the project were to identify nutrition priorities within the community served and implement feasible soup kitchen menu changes that could help the standard menu to better align with those priorities. Here, we describe the key findings and processes used to achieve these goals, which included an initial nutrition needs assessment using soup kitchen guest- and menu-level data, followed by participatory tasting events with soup kitchen guests of new, needs assessment-informed menu items that was complemented by specialized training of kitchen staff to implement new menu items and planning guidelines.

## 2. Methods

### 2.1. Community Setting and Partnership Overview

This project was implemented in Tulsa, Oklahoma, where an estimated 4099 adults and 781 children experience acute or chronic homelessness annually [12]. Located in the city’s downtown district, Iron Gate Tulsa is the largest provider of ready-to-eat meals for unsheltered and unstably housed individuals. In 2019, the organization served 233,122 prepared meals to 7234 unduplicated adults and children. The organization’s mission is, “we feed the hungry and homeless of Tulsa—every day” [13]. At Iron Gate Tulsa, clients are called guests because “we are all guests on this earth and guests treat one another with courtesy, kindness and respect” [13]. Like many nonprofit organizations, its work is supported by a combination of donations and grants. The annual food budget for the meal program is $150,000 (~75% cash and ~25% in kind donations) and meals are prepared by one full-time chef and 5 full-time equivalent (FTE) paid kitchen staff. Volunteer staff also support meal preparation activities (100 unduplicated volunteers, 1700 h per year). The organization has a fully-equipped commercial kitchen and its food production area is approximately 1800 ft^2^.

The “Food is Medicine at Iron Gate” project was led by intervention science public health researchers at the University of Oklahoma (OU) Hudson College of Public Health and culinary arts staff of the OU Culinary Medicine Program in collaboration with executive leadership and meal service staff at Iron Gate Tulsa. The OU Culinary Medicine program was established by the OU-TU School of Community Medicine in 2018 to advance food as medicine initiatives within healthcare and community settings.

We used a data-driven, participatory approach for the planning of this food is medicine initiative. The first phase consisted of a two-part nutrition needs assessment that was followed by a second phase menu standards redesign. These formative planning activities were included in the project timeline to help ensure the final healthy menu re-design would be acceptable for Iron Gate guests, feasible for Iron Gate staff to implement, and financially sustainable. The project’s goals and timeline were designed strategically to advance the organization’s vision for “healthy food access with dignity” under the leadership of the executive director (CVH) and development staff (AS). The project’s academic team members included a professional chef with expertise in culinary medicine (VPC), a Master of Public Health practicum student (LTC), and a registered dietitian with expertise in the food is medicine program design for vulnerable populations (MSW). All activities involving human subject research were reviewed and approved by the Institutional Review Board at the University of Oklahoma Health Sciences Center (Study #12722).

### 2.2. Nutrition Needs Assessment

#### 2.2.1. Surveys

Due to the lack of recently published literature on nutritional risks for people affected by homelessness, we conducted a cross-sectional needs assessment survey to better understand guest health needs, food preferences, dietary restrictions due to dental issues, and overall satisfaction with the soup kitchen menu. After a review of existing instruments, survey questions were initially drafted by two academic members of the team (LTC and MSW) with input and final approval by two community members of the team (CVH and AS). The final survey included 13 standardized questions, with some of these questions allowing for additional open-ended feedback. We assessed health needs using one multi-selection question adapted from the Behavioral Risk Factor Surveillance System (BRFSS) about self-reported chronic health conditions and one question from the Point-in-Time Survey (PIT) about emergency department utilization [14,15]. To better understand the guest’s healthy food preferences, we asked two open-ended questions developed by the team about what favorite vegetables and fruits they would like to see more often on the menu. Due to suspected oral health issues, we adapted two questions from the Oral Health Impact Profile for Edentulous Adults survey to better understand food avoidance behaviors due to teeth, mouth, or denture issues [16]. Guest satisfaction with the current menu was assessed with four, 5-point Likert scale questions designed to measure four dimensions of quality: meeting food preferences, overall taste, meeting health needs, and impact on satiety.

We conducted intercept interviews to collect anonymous survey responses from a convenience sample of walk-up guests on 9 December 2020, prior to the morning meal service. We additionally distributed a self-administered version of the survey on the same day to individuals staying at a temporary overflow shelter, where Iron Gate delivers lunch meals. Surveys were administered by academic team members only, rather than by Iron Gate or shelter staff, to help minimize the coercion to participate and response bias. Researchers remained one to two hours after these two meal services to ensure everyone who wanted to participate had the opportunity. To preserve anonymity, a written statement of the research was provided to all participants in lieu of written consent. Study compensation was mindfully selected to best suit the needs of Iron Gate guests with input from Iron Gate leadership based on items requested most often by guests. Compensation included a blank canvas bag with a fleece blanket, hand-powered flashlight, lip balm with sunscreen, hand sanitizer, and a pair of socks (total value $10).

All survey responses collected through intercept interviews were captured electronically and paper survey responses were entered into the REDCap study database by research staff after their collection [17]. Descriptive statistics were calculated for closed-ended questions using SPSS software, Version 27. Continuous variables were summarized using mean and standard deviation or median and interquartile range, and categorical variables were summarized using frequencies and percentages. Responses to open-ended questions were transcribed as written, reviewed for major themes, and qualitatively summarized. 

#### 2.2.2. Menu Analysis

At baseline, standardized recipes were rarely used by soup kitchen staff for meal production, making traditional menu analysis impossible. Instead, we estimated the nutrition value of meals by weighing the various components of foods served from a random sample of 7 daily menu offerings served to guests over a 12-week period. These daily meal offerings included a hot meal plus a takeaway sandwich. For each daily meal and takeaway selected, an academic member of the research team (LTC) retrieved a prepared meal from the guest meal production line, photographed the meal, and then separately weighed and recorded food items using a digital food scale (My Weigh KD-7000, BBK Tobacco and Foods, LLP, Phoenix, AZ, USA). Kitchen staff were consulted to better understand brand names and types of meats used when needed (e.g., percent lean for beef products). The recorded weight of each food item was entered into Nutritionist Pro, Version 7.8 nutrition analysis software to estimate the nutrition profile of each meal. 

Using SAS Version 9.4, we used the Shapiro–Wilk test in addition to visual inspection of the Q-Q Plots for each nutrient to test whether values were normally distributed across the seven daily menu offerings. We reported the mean and standard deviation for nutrient values that were normally distributed, and the median and interquartile range for nutrient values that were not normally distributed. Macronutrient and micronutrient composition of the standard menu were then compared against the Recommended Daily Allowances (RDA) or Adequate Intake (AI) values for adult males aged 31–50 to evaluate for adequacy. We further compared nutritional profiles of the standard menu against nutrition guidelines for common self-reported health conditions, including the American Heart Association [18], and requested food items from the guest survey. These findings were then applied to inform recommended menu standards and new recipe development during the second phase of the project.

### 2.3. Participatory Menu Redesign

#### 2.3.1. Core Food Selection

Findings from the Phase I needs assessment, including client- and menu-level data, were reviewed by two members of the OU Culinary Medicine team (MSW and LTC) to inform the selection of candidate food items for integration into the new menu. We first identified specific foods that could help to fill major micro and macro-nutrient gaps (as defined by ≤50% of the RDA being met in the standard menu) with particular emphasis on nutrients that are essential for cardiovascular, mental, or immune health (i.e., potassium, magnesium, vitamins A, D, E, K, dietary fiber, and omega 3 fatty acids). These food items were then reviewed against guest’s stated food preferences, food cost, and feasibility (including procurement and preparation) to make final core food item selections. The culinary medicine chef (VPC) then designed recipes featuring core food items in collaboration with the dietitian (MSW), who also developed new menu standards for daily or weekly incorporation of these new core foods.

#### 2.3.2. Participatory Taste Testing

**Figure 1:**
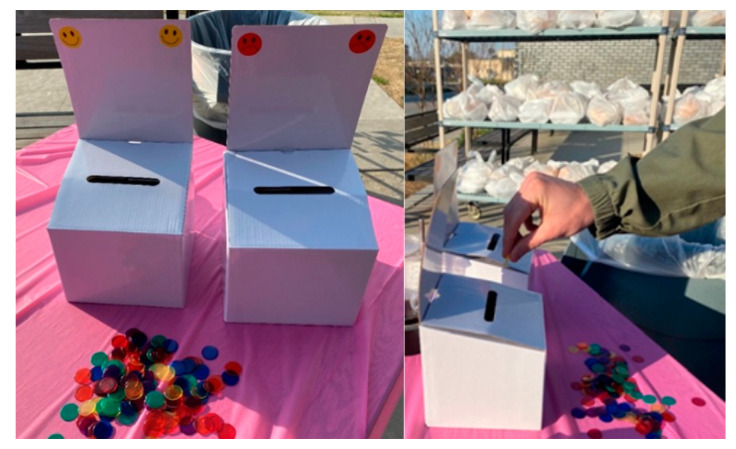
Voting box with tokens used for participatory taste tests.

We conducted taste testing of eight candidate recipes on a semi-weekly basis between 5 March 2021 and 16 June 2021. We additionally taste-tested low-sodium vegetable juice as a high potassium vegetable serving option out of consideration for guests with difficulty chewing vegetables. Recipes were prepared the day before by Culinary Medicine staff on site at Iron Gate. Food items were mostly sampled using 2 oz. souffle cups to promote efficient tasting and to avoid slowing down the serving line. Samples were laid out on a table placed just before guests picked up their drinks and meals. At the end of the table, we placed two opaque voting boxes where guests could use a token to vote whether they liked the test item or not. Each voting box was labeled with a smile face sticker and frown face sticker to clearly delineate a positive or negative response (Figure 1). After each meal service, token votes in each box were counted by an academic member of the team and recorded.

#### 2.3.3. Staff Training

In tandem with ongoing recipe testing and development for new menu standards, the culinary medicine chef conducted four trainings with the Iron Gate kitchen staff. Trainings were required for all kitchen staff, which included the Director of Culinary Services, cooks, the kitchen coordinator, and kitchen stewards. Trainings lasted between 60 and 90 min each. Apart from the head chef (Director of Culinary Services), no other kitchen staff had formal culinary arts training. Additionally, all Iron Gate staff attended an online presentation to learn more about needs assessment findings upon conclusion of the Phase I needs assessment and an in-person orientation to the new recommended menu guidelines upon conclusion of Phase II. These sessions were led by the registered dietitian.

## 3. Results

### 3.1. Phase 1 Nutrition Needs Assessment Survey Results

We collected 114 surveys through the nutrition needs assessment, which included 66 interviewer-administered surveys and 48 self-administered surveys. Of these, one paper survey respondent indicated they were under the age of 18, which was discarded due to being underage for the study. Another paper survey was returned blank, resulting in 112 surveys that were included in the analysis.

#### 3.1.1. Demographics

Respondents were primarily male (66.4%) and ranged in age between 20–71 years, with most being middle-aged (M = 45, SD = 11.9) (Table 1). Half identified as non-white, with people who identified as Black or African American (14.3%) and American Indian or Alaskan Native (9.8%) being the most frequent non-white racial/ethnic identities reported (Table 1).

#### 3.1.2. Health Needs

The most frequent health conditions reported by soup kitchen guests were depressive disorders (50.9%) and cardiovascular diseases (49.1%), followed by diabetes (12.4%), kidney insufficiency/disease (11.6%), and stroke (10.7%). More than one-third of guests (37.6%) reported having multiple chronic health conditions. Results also showed that, among individuals with a chronic health condition, more than one in five (21.8%) reported that their physician recommended a special diet. Diet recommendations related to cardiovascular health (DASH or low-sodium diet) were the most frequently reported (45%). Additionally, half of respondents reported using the emergency room for basic medical care in the past year (Table 2). 

#### 3.1.3. Food Needs, Preferences, and Satisfaction with Current Menu

About one in five (19.6%) respondents reported having a food allergy; however, most hand-written responses when asked to describe the allergy suggested foods primarily associated with food intolerance or preference rather than true food allergy. Milk (n = 6) and eggs (n = 4) were the most common food allergies reported (Table 2). Dental issues that caused avoidance of certain foods were commonly reported (43.1%) with hard fruits (30.4%), raw vegetables (25%), and meat (20.7%) being the most frequent types of foods avoided.

While a majority of respondents reported satisfaction with the menu, several opportunities for improvement were identified, including more offerings that meet food preferences and improved taste of meals (Table 3). Open-ended follow-up questions that asked for suggestions on how meals could be improved included requests for more variety, increased flavor, and wanting more fruits and vegetables. 

Respondents named a wide variety of fruits and vegetables that they wanted to see included in the menu more often. The most frequently requested fruits included oranges (23.2%), bananas (18.9%), and peaches (16.8%). The most frequently requested vegetables included broccoli (25.5%), green beans (20.6%), and carrots (13.7%). Very few respondents indicated they did not like any vegetables (n = 3) or fruits (n = 4) (Table 3).

### 3.2. Phase I Nutrition Analysis of Standard Menu Results

Meals typically included one fruit (banana, orange, or melon). A small iceberg lettuce salad accompanied most meals. Main entrées primarily consisted of a white rice or white noodle base combined with a meat topping (primarily beef) and minimal vegetables (e.g., potato, beans, mixed vegetables, tomatoes). The second meal from each daily entrée was a sandwich on white bread containing either peanut butter or deli meat with cheese. A daily dessert was also included (e.g., cinnamon roll, frosted brownie). Across all meals analyzed, 100% whole grain foods and dark green leafy vegetables were absent.

The average calorie count calculated from the seven randomly sampled daily sets of meals was 1730 calories (865 kcal/meal). Although daily menu offerings supplied nearly a full day’s worth of energy requirements, the content of dietary fiber, vitamin A, vitamin E, vitamin K, vitamin D, magnesium, potassium, and omega 3 essential fatty acids were relatively low (Table 4). Much like the standard American diet, sodium and saturated fat exceeded American Heart Association (AHA) guidelines for cardiovascular health. The average daily menu supplied 11.7% kcal from saturated fat (as compared to the <6% kcal from saturated fat AHA guideline) and the average sodium content exceeded 2300 mg/day [18].

### 3.3. Phase II Menu Redesign

#### 3.3.1. Energy Bites

Informed by needs assessment findings, the OU Culinary Medicine team developed custom recipes for “energy bites” in a variety of flavors including Peanut Butter Chocolate Chip, Blueberry Muffin, Chocolate Chip Oat Muffin, Lemon Chia, Chewy Chocolate Granola, Banana Bread, and Pumpkin Pie. These shelf-stable, easy-to-chew, portable snacks included ingredients to address key nutritional gaps identified in the standard menu, such as chia seeds and flax seed meal (omega 3 fatty acids, dietary fiber) and dried fruit (potassium) as well as the option to add supplemental vitamin D drops to the recipe mix. On average, these “energy bites” supply 7 g of fiber, 108 mg of magnesium, 428 mg of potassium, and 1701 mg of omega 3 (alpha linolenic) fatty acids per 332 kcal serving. Since these bites were intended to promote satiety between meals or for use as a light meal replacement, all were created to be shelf-stable and portable. These recipes provided the additional advantage for potential use as a designated soup kitchen volunteer activity, since each recipe can be batch prepared in about an hour with small teams of 2–3 with oversight by trained kitchen staff.

#### 3.3.2. Nutrition Standards

**Figure 2:**
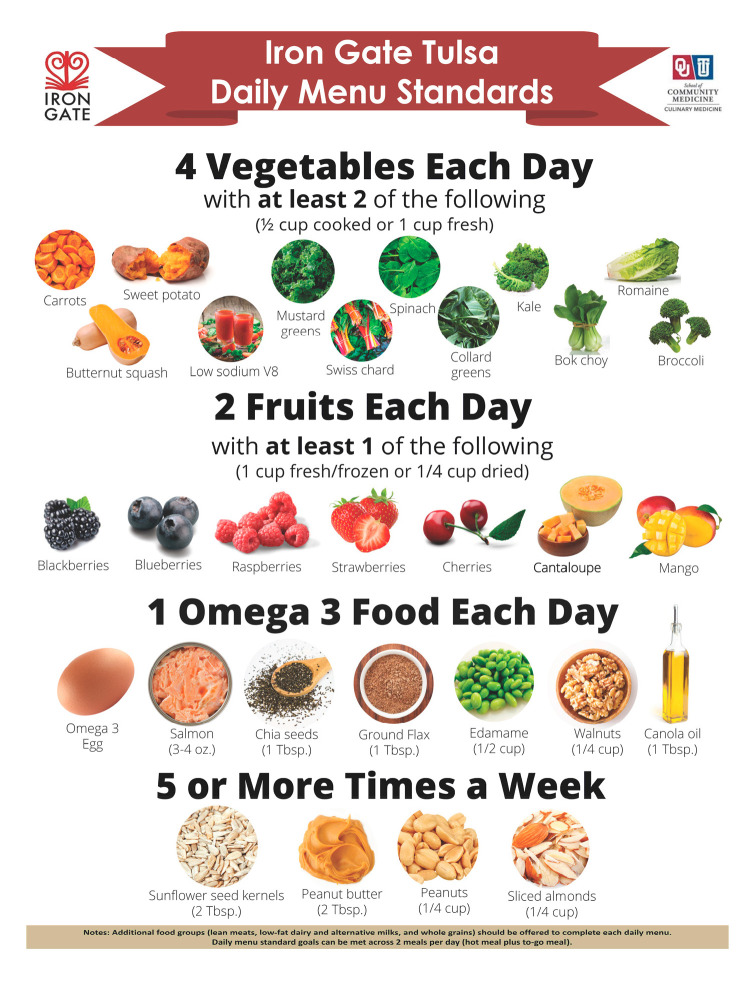
Supplemental food-based recommendations for daily soup kitchen menu.

Additionally, needs assessment findings were applied to create a new set of nutrition standards to help guide kitchen staff in daily meal production. Since the soup kitchen relies partly on donated inventory, menu standards were chosen over more structured cycle menus. Standards were created to help maximize the micronutrient density, essential fatty acid, and fiber of daily meals through a more targeted selection of purchased foods, while allowing for some flexibility in the types of entrées served and use of donations. Between each daily set of served meals, standards encourage a variety of at least four vegetables per day with half being orange or dark green varieties, two fruits per day with an emphasis on berries and beta-carotene rich fruits; an omega 3 food once daily, and use of nuts or seeds at least five times per week with an emphasis on economical, easier to chew varieties (Figure 2).

#### 3.3.3. Participatory Taste Testing

Overall, guests responded positively to example recipes that incorporated various aspects of the new menu standards. All taste tests received over 75% approval, which exceeded satisfaction ratings of the standard menu collected during the Phase 1 needs assessment. Since taste testing occurred over several months, participation rates ranged widely (n = 54–104). Three of the newly-developed energy bites were also tested during this process; satisfaction ranged from 86.9% to 98.5% (Table 5).

#### 3.3.4. Kitchen Staff Training

Although not formally evaluated, kitchen staff training sessions were well attended. Anecdotal feedback shared by the head chef included a greater sense of camaraderie and cooperation among kitchen staff as a result of the additional training sessions. Upon course completion, additional training needs identified by the culinary medicine chef trainer included additional time to: (1) teach specific cooking methods, such as roasting, grilling, sautéing, broiling, steaming, stewing, and braising; (2) practice knife skills and batch preparation techniques for large donations of fresh produce; and (3) address nutrition literacy related the use of ingredients as it relates to the final salt, sugar, and fat content of the finished dish.

## 4. Discussion

This study describes our participatory efforts to identify and best respond to the complex nutrition needs of a nutritionally vulnerable population receiving prepared meals from a modestly resourced soup kitchen provider. We aimed to better understand the unmet nutritional needs of the study population, and in doing so, have contributed to the sparse literature describing the nutrition epidemiology of people without stable housing who rely on soup kitchens. In partnership with our community partner and based on direct input from soup kitchen guests, we also identified strategic menu guidelines that could correct many unmet nutrition needs through the addition of more vegetables, fruits, omega 3 foods, and nuts/seeds. Overall, this study re-confirms the need for well-designed and resourced community-based food assistance programs that can connect more people with healthy dietary essentials. Based on the final outcomes of our research process, we also identified several key recommendations for soup kitchens to consider when preparing for a “food is medicine” menu redesign.

Our nutrition needs assessment suggests that among this community-based sample of people accessing soup kitchens, most individuals have one or more nutrition-related chronic health conditions that may be either worsened or ameliorated by the foods they receive. In this convenience sample, self-reported mental health and cardiovascular diseases were most prominent. This finding is consistent with other research that finds high cardiometabolic risks [19] and mental health disparities [20] among homeless adults. These data provide additional rationale for soup kitchens to transition away from menus that contain ultra-processed foods towards those that include more minimally processed, predominantly plant-centered food items [21,22,23]. The nutrition analysis of the baseline menu identified multiple micronutrient gaps and an imbalance of food groups served, which is consistent with observations from similar studies [4,5]. Satisfaction surveys indicated that many guests enjoy eating and wanted to receive more fruits and vegetables at mealtimes. Recipes prepared with higher fiber, minimally processed ingredients were also well received. Therefore, this study strongly supports the argument that many people affected by homelessness want to eat healthy foods, but are bound by the options available to them [24]. We do not believe this finding is unique to our study population. Other studies examining the readiness for health behavior change among people affected by homelessness found that while most reported low fruit and vegetable consumption (56–66%), the vast majority wanted to improve their intake (74–66%) [25,26], and qualitative research further supports this position [2]. Soup kitchens are often the only reliable source of food for people experiencing homelessness, which suggests that they might function as the determining catalyst in movement from contemplation/preparation to action stages of behavior change.

Although new menu standards were designed to be as simple as possible to apply, soup kitchen providers may still face several key implementation challenges. The implementation of nutrition standards will likely require reliable and affordable healthy food procurement options, kitchen staff with the skills needed to prepare fresh and other minimally processed foods, and the resources (personnel and time) to routinely engage with clientele to ensure menu standards are being applied in acceptable ways. At minimum, these study findings support soup kitchen efforts that prioritize the incorporation of more fresh or frozen fruits and vegetables into daily menus. This change could not only help to improve the overall nutrient density of meals, but also possibly improve potassium: sodium profiles of menus [27]. Although not confirmed in this study, high dietary intake of sodium is common among unsheltered individuals and contributes to uncomfortable side effects reported by this population, such as bloating, in addition to high blood pressure [28,29]. Due to the frequent occurrence of dental issues in this population, culinary staff should be trained on strategies for preparing fruits and vegetables that are easier to chew, such as finely shredding, blending, mashing, and even possibly juicing techniques. Although purchased produce can be costly, food banks routinely receive large donations of fresh fruits and vegetables [30] that are available to partner agency soup kitchens at no to little cost. Thus, these foods could be integrated into volume cooking so long as kitchen staff have foundational cooking literacy in the healthy and tasty preparation of these foods.

This study has important limitations. First, nutrition needs assessment survey responses were collected at a single point in time from a convenience sample of guests accessing food prepared by a specific meal provider; thus, results may not be representative of the entire population of individuals accessing soup kitchens or experiencing homelessness. We further relied on self-reported health information rather than attempting to obtain objective measures of health and nutrition status. Given that little research has been conducted to fully describe the nutrition epidemiology among people affected by homelessness, future research could focus on the comprehensive collection of objective health, nutrition biomarkers (e.g., dermal carotenoids) and dietary assessment data (e.g., 24-h food recalls) to better estimate the prevalence of chronic health conditions and the proportion of people within the population who have insufficient dietary intakes relative to target nutrient or food group values [31]. Finally, the nutrition analysis of the menu reflected a standard portion of meals provided and does not reflect instances where a guest could obtain additional servings from the menu or other nutrients people may obtain outside of the soup kitchen provider.

## 5. Conclusions

In our study, soup kitchen guests preferred more healthy options, reported a wide range of fruits and vegetables they enjoyed, and responded very positively to a healthy menu redesign informed by the “food is medicine” concept. Although we also identified the need for more whole grains and sources of omega 3 fatty acids in soup kitchen menus, we believe integrating more fruits and vegetables is an important first step towards aligning soup kitchen offerings with both the perceived wants and needs of guests. Given the well-established mental and physical health co-morbidities affecting people without housing [31,32], it is imperative for charitable food providers to evaluate the implications of nutrition deficiencies in this population. Policymakers, philanthropic donors, and homelessness service providers should embrace nutrition equity initiatives that seek to better support the food needs of unhoused persons. Soup kitchens and shelters may understandably have concerns about the costs and capacity demands associated with healthier menus, but these types of initiatives may also open doors to new funding opportunities and collaborations. We are hopeful that basic technical assistance training of soup kitchen staff can maximize the uptake and utilization of the 2.2 billion pounds of fresh produce that is available annually to partner organizations through the Feeding America network [32]. Future research should explore the long-term feasibility and other implementation challenges of “food is medicine” guidelines among soup kitchens of different sizes and geographic locations, the impact of technical assistance in overcoming those challenges, and how improved nutritional quality of meals impact the mental and physical wellbeing of soup kitchen guests. Any effort to integrate quality nutrition in ways that align with guests’ wants and needs is an essential first step in achieving nutrition equity for one of the world’s most nutritionally vulnerable groups.

## Figures and Tables

**Table 1:** Demographic Summary.

Characteristic	n (%)
**Sex (n = 107)**	
Female	36 (33.6)
Male	71 (66.4)
**Race/ethnicity (n = 112)**	
White	56 (50)
Black or African American	16 (14.3)
American Indian or Alaskan Native	11 (9.8)
Hispanic/Latino	8 (7.1)
More than one race	10 (8.9)
Other	4 (3.6)
Declined	7 (6.3)
**Age, years (n = 109), M (SD)**	45 (11.9)

**Table 2:** Self-reported health needs as reported in nutrition needs assessment survey among people accessing food at a soup kitchen.

Characteristic	n (%)
**Emergency room use (12 mos.) (n = 110)**	
% Yes	55 (50)
**Chronic illnesses (n = 112)**	
Depressive disorder	57 (50.9)
Cardiovascular disease ^1^	55 (49.1)
Diabetes/Pre-diabetes	12 (12.4)
Kidney insufficiency/failure	13 (11.6)
Stroke	12 (10.7)
**Special diet recommended by provider (n = 110)**	
% Yes	24 (21.8)
**Food Allergies (n = 112)**	
% Yes	22 (19.6)
**Avoid food due to mouth, teeth, or gums (n = 109)**	
% Yes	47 (43.1)
**Types of food avoided**	
Hard fruits	28 (30.4)
Raw vegetables	23 (25)
Other	22 (23.9)
Meat	19 (20.7)

^1^ Cardiovascular disease included hypertension, high blood pressure, and heart attack.

**Table 3:** Most requested fruit and vegetables and meal satisfaction ratings at baseline.

Characteristic	n (%)
**Most requested fruits (n = 95)**	
Oranges	22 (23.2)
Bananas	18 (18.9)
Peaches	16 (16.8)
**Most requested vegetables ^1^ (n = 102)**	
Broccoli	26 (25.5)
Green beans	21 (20.6)
Carrots	14 (13.7)
**Meal satisfaction**	
Meets food preferences, % often/always (n = 108)	73 (67.6)
Taste, % satisfied/very satisfied (n = 106)	69 (65.1)
Healthfulness, % satisfied/very satisfied (n = 107)	77 (72.0)
Satiety, % very filling/extremely filling (n = 106)	76 (71.7)

^1^ One quarter of respondents (n = 26, 25.5%) wrote “corn” as a preferred vegetable; this was not included in the top 3 vegetables reported due to corn being a grain.

**Table 4:** Nutrition profile of standard baseline menu averaged from 7 daily menu offerings.

Nutrient	M (SD)	% DV ^1^	Range
**Macronutrients**			
Calories (kcal)	1729.93 (254.40)	78.63	1318.69–2114.54
Protein (g)	68.84 (22.99)	122.93	46.30–107.14
Carbohydrates (g)	230.97 (20.21)	177.67	199–259.38
Dietary Fiber, total (g)	18.11 (5.87)	58.42	11.38–28.36
Sugar, total (g)	64.58 (24.18)	--	33.5–105.38
Fat, Total (g)	61.74 (17.76)	83.37–126.28 ^2^	39.18–84.73
Cholesterol (mg) ^3^	185.55 (87.70)	61.67	72.12–345.34
Saturated Fat (g)	22.46 (6.32)	91.90 ^2^	11.42–29.64
Trans Fatty Acid (g) ^3,4^	0.66 (0.45)	--	0.46–1.59
Monounsaturated Fat (g)	17.69 (7.72)	--	10.49–28.99
Polyunsaturated Fat (g)	7.91 (3.65)	--	4.92–15.03
Omega 3s (PFA18:3, Linolenic) (g)	0.63 (0.29)	39.38	0.31–1.12
**Minerals**			
Sodium (mg)	2435.81 (1051.15)	105.9	1333.32–4498.78
Calcium (mg)	597.40 (310.85)	59.74	159.37–914.39
Iron (mg)	12.95 (3.41)	161.88	9.22–18.02
Magnesium (mg)	189.75 (73.43)	45.18	118.5–301.89
Phosphorus (mg)	899.12 (401.71)	128.45	387.67–1424.89
Potassium (mg)	1627.74 (323.64)	47.87	1299.88–2250.83
Zinc (mg)	9.39 (5.43)	85.36	4.4–19.41
**Vitamins**			
Vitamin A (RAE)	267.14 (144.72)	29.69	46.04–511.26
Vitamin E (mg) ^4^	0.25 (0.38)	3.93	0.14–2.47
Vitamin K (mcg)	30.56 (13.13)	25.47	6.36–47
Vitamin D (IU) ^4^	24.28 (20.38)	4.74	2.38–87.29
Vitamin C (mg)	89.50 (41.31)	99.44	51.82–162.37
Thiamin (mg)	1.52 (0.26)	126.67	1.23–1.95
Riboflavin (mg)	1.19 (0.23)	91.54	0.88–1.5
Niacin (mg)	16.79 (3.39)	104.94	12.98–23.69
Folate, total (mcg)	351.81 (54.00)	87.95	274.83–406.14
Vitamin B6 (mg)	1.28 (0.32)	98.46	0.78–1.82
Vitamin B12 (mcg)	2.90 (1.82)	120.83	0.86–6.37
Choline (mg)	154.40 (63.04)	28.07	56.62–248.53

^1^ % of RDA for adults each nutrient using males aged 31–50 as reference group; ^2^ based on an Acceptable Macronutrient Distribution Range (AMDR) of 20% to 35% for a 2200 kcal diet; ^3^ National Academies recommends that trans-fat and dietary cholesterol consumption be as low as possible without compromising the nutritional adequacy of the diet; ^4^ data were not normally distributed, Mdn (IQR) displayed instead of M (SD).

**Table 5:** Taste Testing Results.

Item Tested	Total Samples Distributed (n)	% Voting Yes to Include in New Menu
**Selection of Energy Bites**		
Pumpkin spice	84	86.9
Chocolate muffin	81	91.4
Peanut butter cup	67	98.5
**Other New Potential Menu Items**		
Low sodium vegetable juice	104	76.9
Spinach with vinaigrette	60	78.3
Kale salad with apple vinaigrette	60	80.0
Eggplant ratatouille	69	84.1
Sweet potatoes, roasted	84	92.9
Smoky black bean chili	54	100

## Data Availability

The data presented in this study are available on request from the corresponding author.
